# Interaction study of monoisoamyl dimercaptosuccinic acid with bovine serum albumin using biophysical and molecular docking approaches

**DOI:** 10.1038/s41598-021-83534-0

**Published:** 2021-02-18

**Authors:** Ashima Thakur, Jayant Patwa, Suyash Pant, Abha Sharma, S. J. S. Flora

**Affiliations:** 1 Department of Medicinal Chemistry, National Institute of Pharmaceutical Education and Research, Raebareli, Lucknow, UP 226002 India; 2 Department of Pharmacology and Toxicology, National Institute of Pharmaceutical Education and Research, Raebareli, Lucknow, UP 226002 India; 3 Department of Pharmacoinformatics, National Institute of Pharmaceutical Education and Research, Kolkata, India

**Keywords:** Biophysical methods, Optical spectroscopy

## Abstract

Monoisoamyl 2,3-dimercaptosuccinic acid (MiADMSA), a lipophilic chelator has been evaluated for its potential use as an antidote in arsenic poisoning. The pharmacokinetics and pharmacodynamics properties of a drug could be understood via study its mechanism of interaction with bovine serum albumin protein (BSA). Therefore, the interaction between MiADMSA with BSA was investigated using various spectroscopic techniques and computational methods. Linear quenching of BSA intrinsic fluorescence intensity with the increasing concentration of MiADMSA was observed in the fluorescence study. Furthermore, synchronous results revealed that MiADMSA slightly changed the conformation of BSA. The binding constant value of the BSA-MiADMSA complex was found 1.60 × 10^4^ M^−1^ at 298 K. The value of thermodynamic parameters ΔG, ΔH, and ΔS described that the process is spontaneous, endothermic, and hydrophobic forces are involved in the interaction of MiADMSA with BSA. Competitive site marker experiments showed that MiADMSA binds to site-II of BSA. Conformational changes of BSA with the interaction of MiADMSA were apparent by CD, UV–Visible, FT-IR, and 3D fluorescence spectroscopy. To strengthen the experimental findings we have also performed a theoretical study on the BSA-MiADMSA complex. Two sites were identified with docking score of − 6.642 kcal/mol at site II_a_ and − 3.80 kcal/mol for site II_b_ via molecular docking study. Molecular dynamics simulation study inferred the stability of the BSA-MiADMSA complex which was analyzed in a long simulation run. The experimental and computational studies have shown the effective binding of MiADMSA with BSA which is essential for the transportation and elimination of a drug from the body.

## Introduction

**Figure 1:**
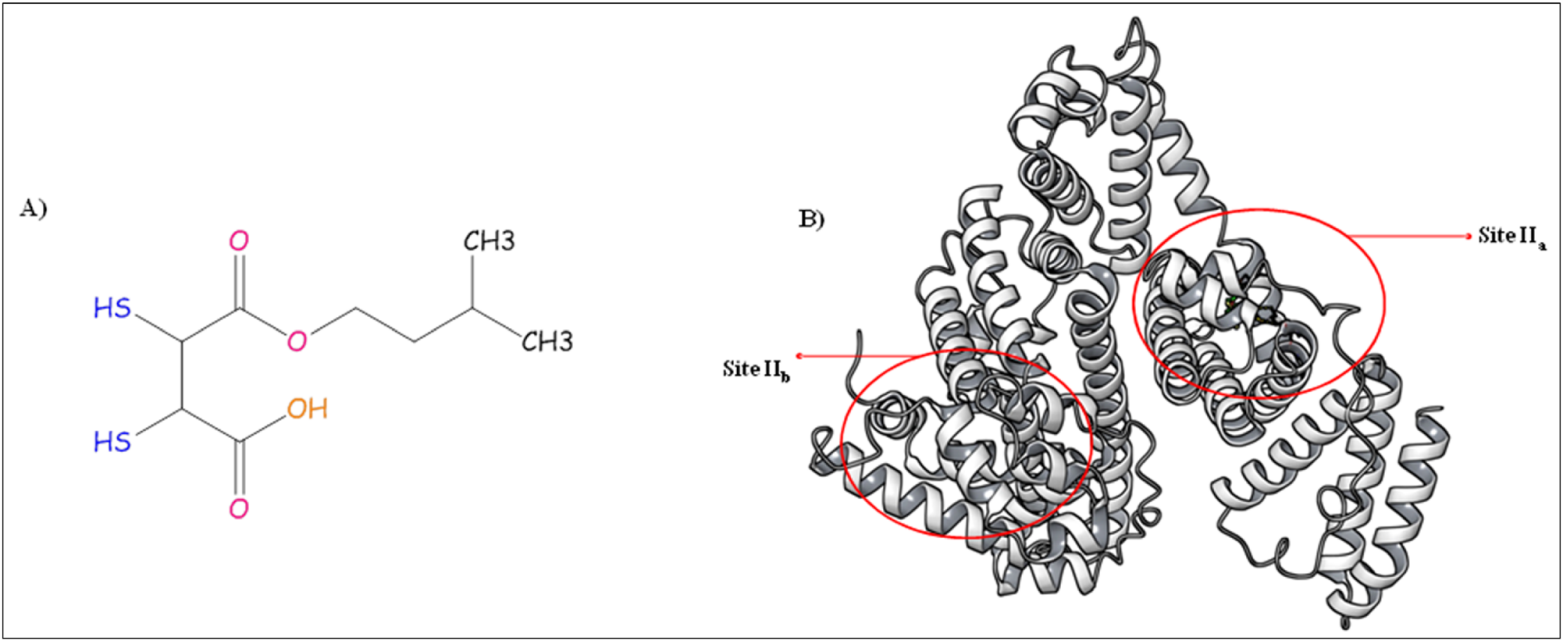
(**A**) Chemical structure of MiADMSA (Chem BioDraw Ultra, 14.0) (**B**) Crystal structure of BSA (PDB ID: 4F5S) with two possible binding sites (Desmond Academic license 2018-4, https://www.schrodinger.com/citations#Desmond).

MiADMSA (Fig. 1A) is a monoester of dimercaptosuccinic acid (DMSA), synthesize by the esterification of DMSA with isoamyl alcohol in a controlled condition^1^. Literature reports suggested that MiADMSA is an effective antidote against heavy metal poisoning. It is a lipophilic thiol chelator that has completed Phase I clinical trial and gaining recognition as a potential antidote for use in the treatment of chronic arsenic poisoning^2,3^. The chelation property of MiADMSA could be attributed due to the presence of thiol moiety in the structure^1,4^. Once the drug gets absorbed and reaches into the systemic circulation, it interacts with various plasma proteins such as serum albumin, globulin alpha − 1 acid glycoprotein, etc. Among them, albumin is abundantly present in the blood and plays an important role in drug transportation^5–8^. Interaction of drug and serum albumin may affect the pharmacokinetic parameters of the drug and eventually can alter the therapeutic effects of the drug. Therefore, the investigation of the biological interactions of MiADMSA with albumin is critically important.

Bovine serum albumin (BSA) (Fig. 1B) is structurally homologous to human serum albumin (HSA) and is considerably cost-effective. BSA has a sequence identity of more than 72% and a amino acids similarity of more than 83% to HSA^9,10^. X-ray crystallography of BSA suggests that it has 583 amino acid residues and three homologous domains (I, II, III), which further splits into two helical subdomains A and B^11,12^. The subdomain IIA and IIIA are designated as site I and site II, which are considered as a major binding site for the molecules. The fluorescence property of the BSA could be attributed due to the presence of tryptophan residue in the structure^13,14^. There are two tryptophan residues in BSA, located in subdomain IIA and IA at positions 212 and 134, respectively. The chemical microenvironment of Trp 212 of BSA is equivalent to Trp 214 in human serum albumin (HSA)^15,16^. Herein, we have planned to investigate the mechanistic interactions between BSA and MiADMSA under physiological conditions using different spectroscopic techniques UV–Visible spectroscopy, fluorescence spectroscopy, infrared spectroscopy, and circular dichroism (CD). The site of binding of the MiADMSA was investigated using molecular docking and a dynamic approach.

## Materials and methods

BSA was obtained from HiMedia Laboratories (Mumbai, India) and used without any further purification. Ranitidine, Ibuprofen, sodium phosphate dibasic, and monobasic were procured from Sigma Aldrich (St Louis, Missouri, USA). MiADMSA (Purity 99.98%) was received as a gift sample from Cadila Pharma, Ahmedabad, India. The solutions were prepared in Millipore water.

### Sample preparation

Stock solutions of BSA (1 mM) and MiADMSA (1 mM) were prepared by using a phosphate buffer pH 7.4 (pH adjusted using NaOH and HCl). The final concentration of BSA (5 µM) was made from the stock solution using phosphate buffer pH 7.4. Different concentration of MiADMSA was prepared from the stock solutions (1 mM) using phosphate buffer pH 7.4.

### Fluorescence emission experiments

All the fluorescence emission spectra were recorded on the Agilent Carry Eclipse fluorescence spectrophotometer (Santa Clara, US) using a 1 cm quartz cuvette. In this study, the excitation wavelength was set at 280 nm and the fluorescence intensity was measured at 345 nm with a slit width of 5 nm. Further, the entire spectra were executed in the range of 250–600 nm for the BSA and BSA-MiADMSA complex. In this experiment, the concentration of BSA was kept constant at 5 µM with varying concentrations of MiADMSA (0–128 µM) at different temperatures (298 K, 303 K, 308 K, and 318 K)^17^. Moreover, Synchronous experiments have been carried out on the same spectrofluorometer. All the synchronous fluorescence spectra were obtained at Δλ = 15 nm (250–320 nm) and Δλ = 60 nm (240–330) at 298 K. The concentration of BSA (5 µM) was fixed with increasing concentrations of MiADMSA (2–64 µM)^18^. A three-dimensional experiment of the BSA and BSA-MiADMSA complex was carried out using a spectrofluorometer.

### UV–Vis absorption spectroscopy study

The study was carried out by titrating BSA (5 µM) with different concentrations of MiADMSA (0–8 µM). The spectrum was recorded on an Agilent Carry Eclipse UV spectrophotometer (Santa Clara, US) using a 1 cm quartz cuvette at 298 K in the range of 200–600 nm and the baseline was corrected^17,19^. MeOH:H_2_O (1:9) mixture was used as a reference solution for UV–Vis studies.

### Competitive site binding studies

The competitive binding study was carried out to evaluate the binding site of BSA occupied by the MiADMSA^20^. The experiments were executed at 298 K by using Ranitidine as a site-I marker and ibuprofen as a site-II marker. Varying concentration of MiADMSA (0–128 µM) was added to the solution containing the same concentration of BSA and site markers (5 µM)^21^. Fluorescence intensity was measured at emission wavelength (λ_em_ = 345 nm) and binding constants values were determined.

### Thermodynamics parameters

The thermodynamics parameters are essential to evaluate the binding interaction between the drug and protein. For the thermodynamics calculations, we have measured the binding constant at different temperatures (298 K, 303 K, 308 K, and 318 K). Gibb’s free energy (ΔG), enthalpy change (ΔH), and entropy change (ΔS) were calculated from the van’t Hoff plot^22^.

### Circular dichroism (CD) spectral measurements

The spectral measurements were carried out in the Jasco CD spectropolarimeter (J-1500, Tokyo, Japan) to determine the structural changes in protein upon binding with MiADMSA. The experiments were recorded in the range of 200–320 nm with a scanning speed of 200 nm/min. and response time of 1 sec. at room temperature. For each spectra baseline was corrected. The concentration of BSA was fixed to 2.5 µM with 25 µM and 50 µM concentration of MiADMSA.

### Fourier transform infrared spectral (FTIR) analysis

Bruker FT-IR spectrometer (Massachusetts, US) instrument with Attenuated Total Reflectance (ATR) mode linked with OPUS software was used. All the spectra were recorded at 24 scans at room temperature (298 K). Background measurement was run before taking the spectra of BSA and BSA with drug complex i.e. MiADMSA. The baseline correction was done for all the samples using OPUS software^23^.

### Protein preparation and molecular docking

All computational calculations were performed in the Schrodinger suite 2018-4. The crystal structure of BSA and equine serum albumin (ESA) was retrieved from the RCSB database https://academic.oup.com/nar/article/28/1/235/2384399^24–26^. ESA (PDB ID: 6U4X)^26^ and BSA (PDB ID: 4F5S)^27,28^ were prepared using Schrodinger protein preparation wizard and all ionizable residues were set to their probable protonation state at pH 7.4 using ProPka^29^. Finally, the corrected structure with all hydrogens added was minimized using the OPLS2005 force field. MiADMSA was drawn in Schrodinger and the geometry was minimized using OPLS2005 force field at pH 7.4 https://www.schrodinger.com/citations.

Binding site analysis was performed using the sitemap module in Schrodinger 2018-4. A glide grid module was utilized to generate a grid within a 5.0-Å radius of ibuprofen for BSA (Arg 409, Arg 406, Arg 410, Ser 488, Tyr 410) and ESA consisting of conserved residues. The size of the grid box was manually adjusted to 18 Å. A glide module was utilized to perform the docking experiment at the desired site and the best pose was taken further for 200 ns of molecular dynamics simulation and MMGBSA binding free energy calculation^17^.

### Molecular dynamic study

All calculations were performed by Desmond package 2018-4 using the OPLS2005 force field. The protein–ligand docked complex was solvated with the single point charge water model and the overall charge of the system was neutralized^30^. A default relaxation protocol of Desmond was used to equilibrate the system followed by 200 ns of the production run^31^. Hydrogen positions were constrained by the M-SHAKE algorithm. Long-range electrostatics were computed every third-time step by using the Smooth particle mesh Ewald method. A 9.0-Å radius cut-off was set for columbic short-range interaction cut off method^32^.

Post simulation analysis was performed to understand the average change in the protein–ligand complex from the initial frame. Changes were recorded in the angstrom unit, fluctuation within 1–3 Å are acceptable for small, globular proteins and above that needs to be analyzed properly. Furthermore, we have also calculated the MMGBSA binding free energy of the simulated complex using the prime module.

## Results and discussion

### Fluorescence study

#### Steady-state fluorescence and quenching mechanism study

Alteration in the intrinsic fluorescence of protein with quencher is helpful in understanding the mechanism of their interaction with protein. BSA is composed of mainly three amino acids Trp, Tyr, Phe in a proportion of 100: 9: 0.5, and majorly fluorescence is due to Trp which has the highest quantum yield^33^. The fluorescence spectra of BSA were recorded at the excitation wavelength (λ_ex_ = 280 nm) and emission wavelength (λ_em_ = 345 nm) with MiADMSA. The gradual addition of MiADMSA in BSA led to a decrease in fluorescence intensity of BSA which might be due to the formation of a complex between BSA and MiADMSA^34^. It is evident from the spectrum that the peak shape up to 32 µM concentrations remained the same and further at 68 µM and 126 µM concentration, a slight change in peak shape was observed. There was a 14 nm change in emission wavelength from 345 to 331 nm at 298 K.

**Figure 2:**
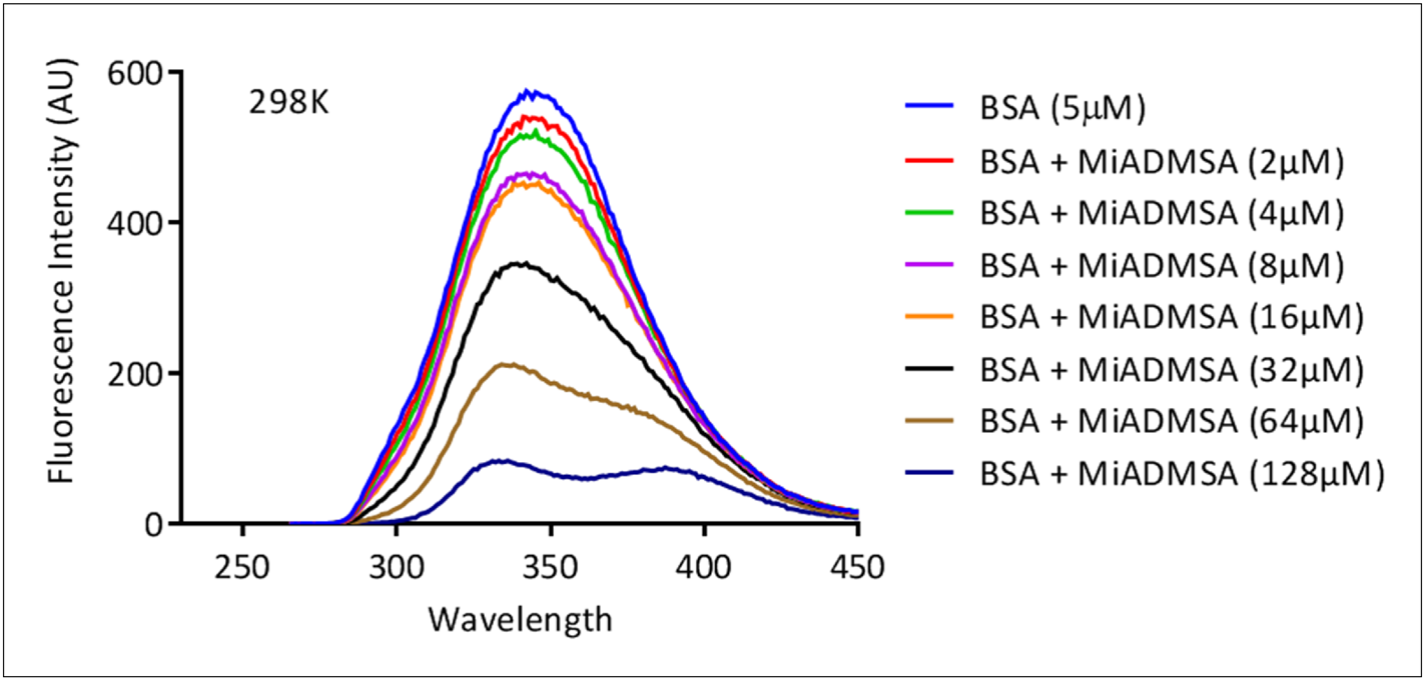
The fluorescence quenching of BSA (5 µM) with increasing concentration of MiADMSA (0–128 µM) at 298 K.

**Figure 3:**
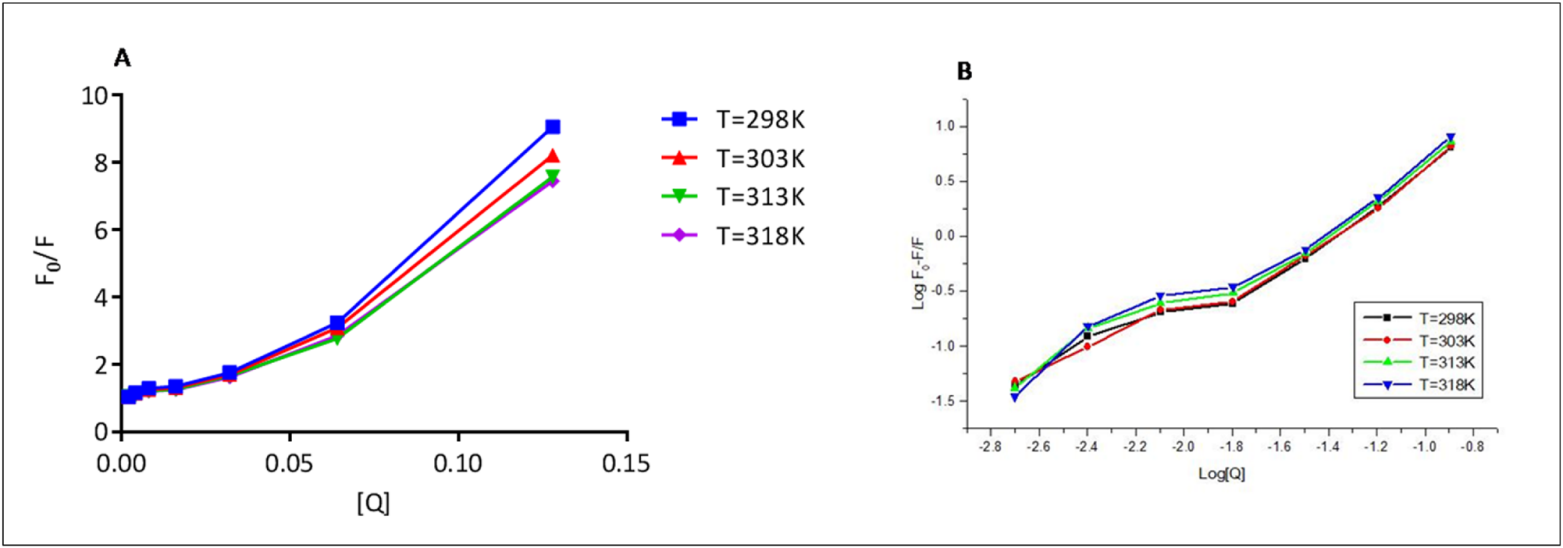
(**A**) Stern–Volmer plot of [F_0_/F] versus [Q] (**B**) Plot of Log [F_0_-F]/F versus Log [Q] at 298 K, 303 K, 308 K, 318 K.

Primarily, fluorescence quenching can be proceeded in two ways- static and dynamic quenching^35^. The two types of the phenomenon may occur during the quenching process i.e., ground-state complex formation and collisional encounters which do decide the type of quenching mechanism. The former process indicates static and the latter one is a dynamic quenching mechanism. The quenching mechanism of ligand–protein interaction was explored by recording spectra at variable temperatures. The change in quenching constant value at different temperature indicates whether quenching is static or dynamic. In the case of static, there is a decrease in quenching constant value with rising in temperature as it causes the ground state complex formation at a slower rate and vice versa^36^. Figure 2 shows the systematic quenching in the fluorescence intensity of BSA in presence of MiADMSA at 298 K (spectra at other temperatures (303 K, 313 K, and 318 K) are given in supplementary file (S1 Fig. 1)). The mechanism of quenching was examined by Stern Volmer Eq. (1) given as below:

1 $${\text{F}}_{{\text{o}}} {\text{/F}} = {1} + {\text{K}}_{{{\text{sv}}}} \left[ {\text{Q}} \right] = {1} + {\text{K}}_{{\text{q}}}\uptau _{0} \left[ {\text{Q}} \right]$$

2 $${\text{K}}_{{\text{q}}} = {\text{K}}_{{{\text{sv}}}} {/}\uptau _{0}$$

where F_o_ and F are the fluorescence intensity of BSA and BSA-MiADMSA complex. “K_sv_” is the stern–volmer constant, Q is the concentration of quencher, “K_q”_ is the bimolecular quenching rate constant, and “τ_0”_ is a standard lifetime of the fluorophore contains Trp, Tyr, and Phe and having a value of 10^−8^ sec^−1^. The value of K_sv_ was obtained from the slope of the graph between [F_o_/F] versus [Q] (Fig. 3A) and the value of K_q_ was determined from Eq. (2). We found an increase in Kq values with a rise in temperature that showed an increase in the diffusion rate of quencher which led to an increase in the collision rate at a higher temperature (Table 1). This experimental result indicates that the fluorescence of BSA in presence of MiADMSA was quenched dynamically.

**Table 1:** Various parameters of measuring the interaction between BSA-MiADMSA.

Temperature (K)	K_sv_ (M^−1^)	K_q_ (M^−1^ sec^−1^)	R^2^	K_b_ (M^−1^)	n	R^2^
298	4.95 × 10^4^	4.95 × 10^12^	0.9476	1.60 × 10^4^	1.1	0.9589
303	5.02 × 10^4^	5.02 × 10^12^	0.9423	1.62 × 10^4^	1.1	0.9662
308	5.53 × 10^4^	5.53 × 10^12^	0.9481	1.69 × 10^4^	1.1	0.9618
318	6.16 × 10^4^	6.16 × 10^12^	0.9433	1.79 × 10^4^	1.1	0.9613

#### Binding sites and binding constant

The binding constant and number of the binding site of the drug in protein were evaluated by the modified Stern Volmer Eq. (3)^37^.

3 $$\frac{{{\text{Log}}[{\text{F}}_{0} - {\text{F}}]}}{{\text{F}}} = {\text{LogK}}_{{\text{b}}} + {\text{n}}\;\log \;\left[ {\text{Q}} \right]$$

where “K_b_” is the binding constant and “n” is the number of binding site

The values of K_b_ and n were calculated from the intercept and slope of the graph plotted between log [F_0_ − F] versus log [Q], respectively (Fig. 3B). The drugs should bind to BSA reversibly for its storage and distribution in the body and the binding constant value lie in the range of 1–15 × 10^4^ M^−1^ indicate the average binding affinity of the drug to BSA and reversible complex formation between the two. In our study, we found the value of K_b_ in the reported range and binding number (n) obtained from the graph at different temperatures is nearly 1. Therefore, based on results obtained from fluorescence experiments, we concluded that there is one binding site in BSA for ligand^38^.

### Synchronous fluorescence analysis

**Figure 4:**
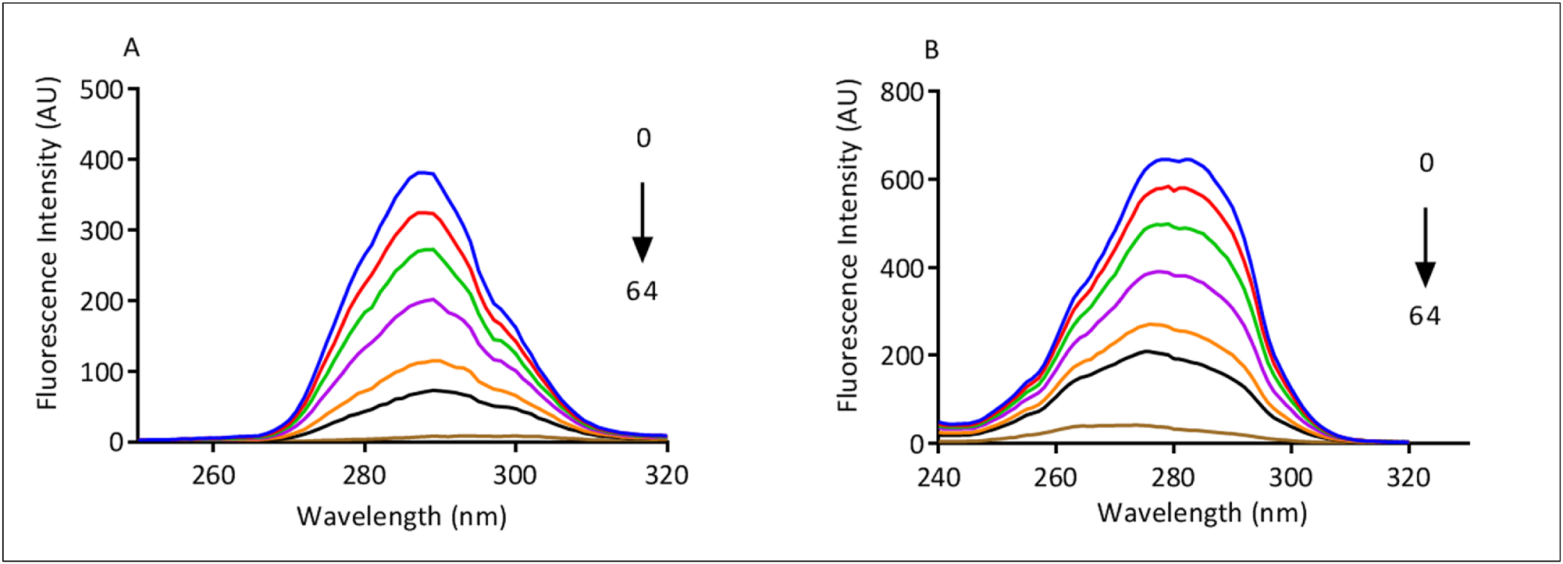
(**A**) Synchronous emission spectra of BSA (5 µM) with MiADMSA (0–64 µM) at Δλ = 15 nm for Tyr (**B**) Synchronous emission spectra of BSA (5 µM) with MiADMSA (0–64 µM) at Δλ = 60 nm for Trp at 298 K.

Further, the interaction between BSA and MiADMSA was investigated using synchronous fluorescence spectroscopy^21,39^. The study is helpful to understand the change in the microenvironment of fluorophore residues mainly Tyr and Trp residues of BSA^40^. The synchronous fluorescence spectra at Δλ = 15 nm and 60 nm of BSA (5 µM) with varying concentrations of MiADMSA (0 to 64 μM) are shown in Fig. 4. The spectral change at Δλ = 15 nm shows that Tyr residue is involved in interaction with drug. No shift in emission wavelength of BSA in presence of MiADMSA at Δλ = 15 nm was observed (Fig. 4A).

The binding of MiADMSA with BSA does not alter the microenvironment around the Tyr amino acid. While Fig. 4B at Δλ = 60 nm a shift in the emission wavelength from 278 to 275 nm was observed which indicates the conformational changes in BSA due to alterations in the microenvironment of Trp amino acid, resulting in an increase in hydrophobicity and decrease in hydrophilicity^18,41^.

### 3D spectroflurometric analysis

**Figure 5:**
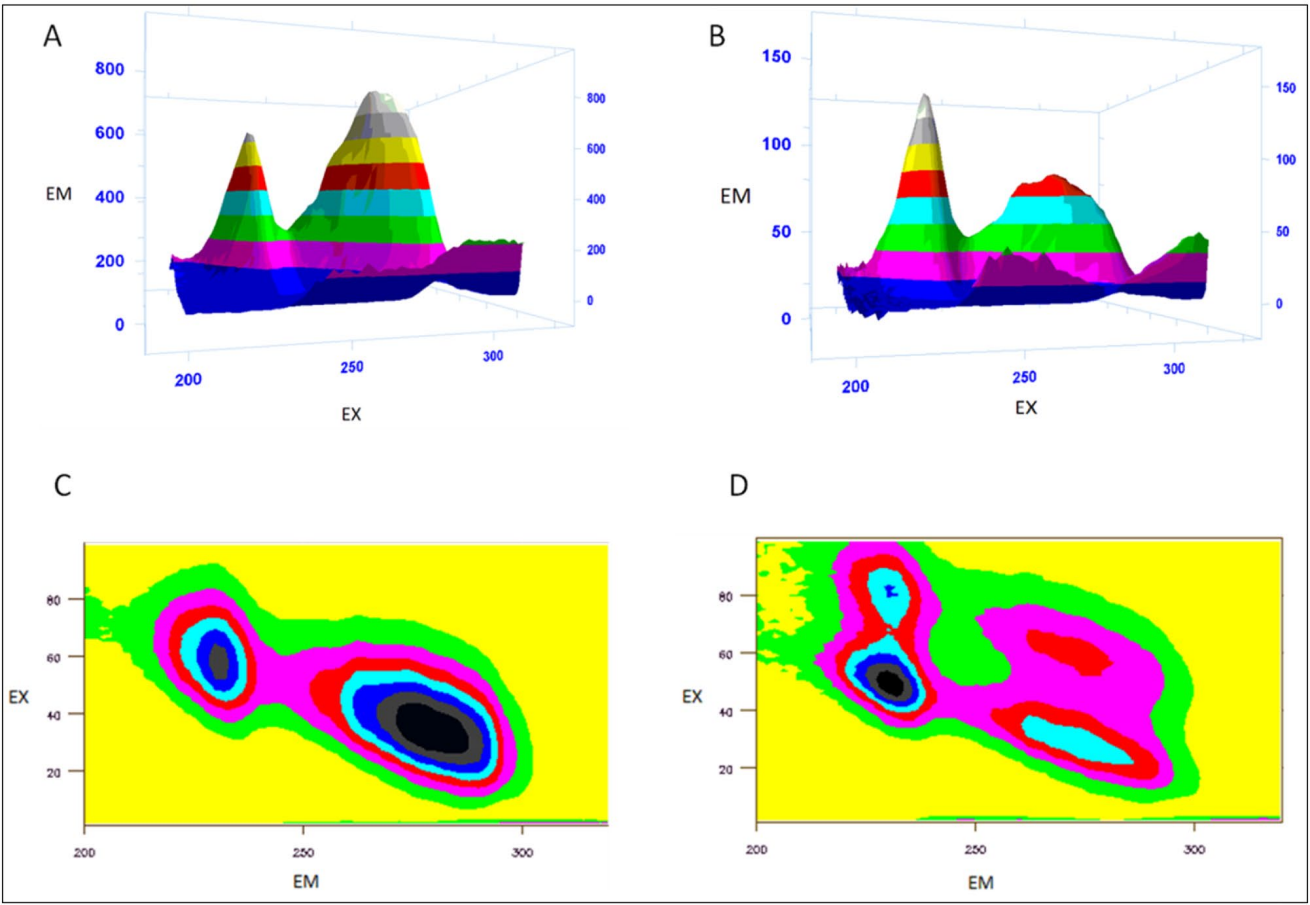
(**A**,**C**): 3D and contour plot of BSA (5 µM). (**B**,**D**) 3D and contour plot of BSA (5 µM) with MiADMSA (25 µM) (Carry Eclipse Win FLR software, version 1.1 (132)).

The 3D spectrofluorometric analysis was carried out to understand the structural changes of BSA in presence of the MiADMSA. The 3D spectrum has shown two characteristic peaks of BSA (5 µM) at an excitation wavelength of 280 nm and an emission wavelength of 340 nm with an interval of 2 nm. The first obtained peak in the 3D illustration might be due to the pi–pi transitions in the polypeptide structure present in BSA while another one might be due to aromatic amino acids present in BSA. Interestingly, the fluorescence intensity was significantly declined upon the addition of MiADMSA (25 µM). The contour plot also represents microenvironmental changes of the BSA in presence of MiADMSA (Fig. 5). The following changes led us to conclude that MiADMSA alters the structural conformational of BSA^36^.

### UV–visible spectral analysis

**Figure 6:**
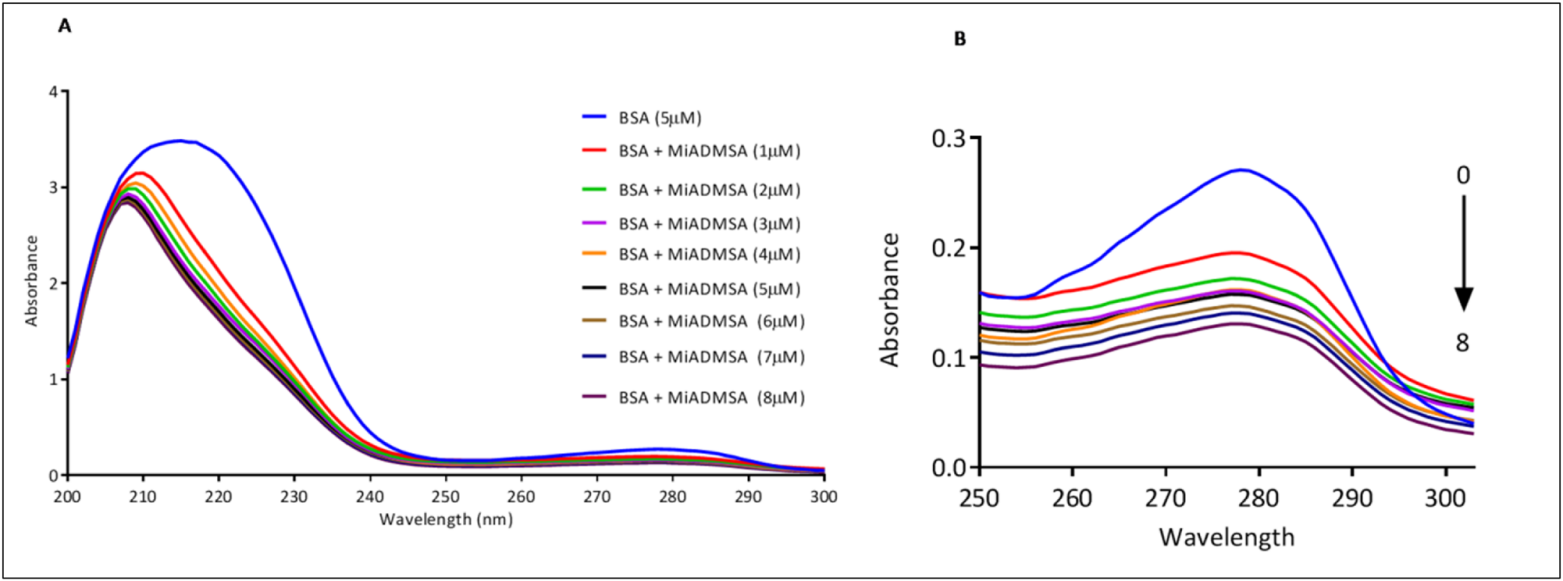
(**A**) UV absorbance spectra of BSA (5 µM) in the presence of MiADMSA (0–8 µM) (**B**) extended view of absorbance spectra of BSA with MiADMSA.

The UV–vis spectroscopy study gives complete information regarding the conformational changes in the structure of protein due to protein–ligand interactions or complex formation between them^42,43^. BSA protein shows an absorption band at λ_max_ 280 nm which is arisen from π–π* electronic transitions of aromatic amino acids^44^. Alterations in the absorption spectrum of BSA on adding up ligand furnish information about the type of changes and their rationale^19^. The hypochromic shift was observed on adding MiADMSA in increasing concentration from 0 to 8 µM which suggests that there is a complex formation between them (Fig. 6)^45^. In the case of static quenching mechanism, there is a ground-state complex formation between the BSA and ligand leading to an increase in the absorbance of BSA^46^. However, we observed a decrease in intensity of the absorption band of BSA upon the increasing concentration of MiADMSA, this could be due to dynamic quenching between MiADMSA and BSA.

### Site selective binding of MiADMSA on BSA

**Figure 7:**
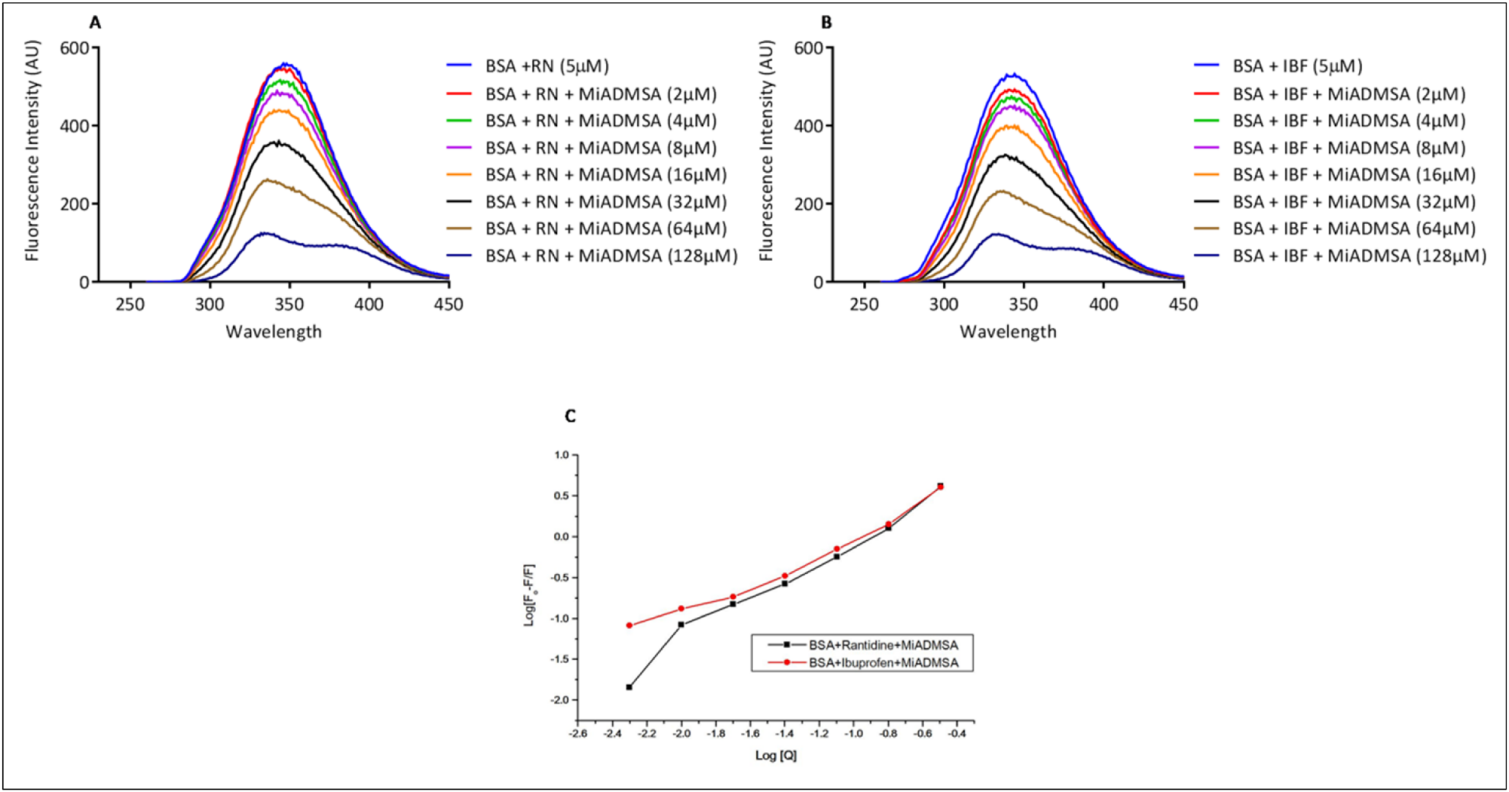
(**A**) Emission spectra of BSA with MiADMSA in presence of site marker Ranitidine; (**B**) Emission spectra of BSA with MiADMSA in presence of site marker Ibuprofen (**C**) Plots of binding constant of BSA with MiADMSA in presence of Ranitidine and Ibuprofen.

BSA protein possesses two binding sites- site-I and site-II and to identify the binding site of MiADMSA in BSA, we carried out competitive binding experiments. Ranitidine and Ibuprofen were used as specific site markers for site-I and site-II of BSA to analyze the binding site displaced by MiADMSA in competition with site markers^22^. The concentration of BSA and site markers was kept the same throughout the experiment with successive additions of MiADMSA (0-128 µM). Thus, displacement of site markers bound to BSA by MiADMSA was measured by fluorescence titrations at an excitation wavelength of 280 nm at 298 K. The addition of the Ranitidine site marker in BSA solution caused quenching of fluorescence intensity with the blue shift from wavelength 345 nm to 333 nm. Then, in the BSA-Ranitidine complex, the incremental addition of MiADMSA solution of 2 µM to 128 µM concentration resulted further decrease in fluorescence intensity indicates that MiADMSA affects the binding of Ranitidine to BSA (Fig. 7A) . The same decrease in fluorescence intensity with shift from 346 to 332 nm for site II on adding MiADMSA in the BSA-Ibuprofen complex was observed (Fig. 7B) . The binding constant value of BSA-MiADMSA in presence of ranitidine and ibuprofen was found 1.16 × 10^4^ M^−1^ and 0.91 × 10^4^ M^−1^, respectively. The change in binding constant values inferred that there was more alteration in site-II of BSA as compare to site-I^47^.

### Thermodynamics calculations

**Figure 8:**
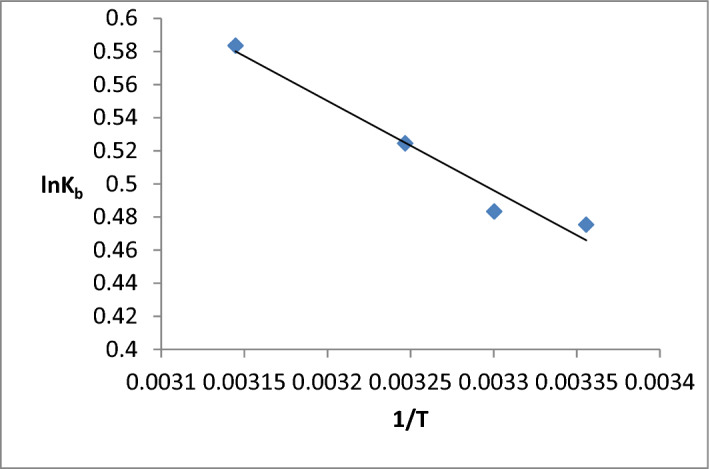
Van’t Hoff plot for binding of BSA with MiADMSA.

The binding of drugs or ligands with protein usually involved four types of forces that include hydrogen bonds, Van der Waals, electrostatic and hydrophobic interactions^22^. To find out the types of forces acting during binding of ligand and protein, various thermodynamics parameters such as entropy change [ΔS], enthalpy change [ΔH], and Gibb's free energy [ΔG] were calculated from van’t Hoff plot using Eq. (4):

4 $${\text{LogK}}_{{\text{b}}} = - \Delta {\text{H}}/{2}.{3}0{\text{3RT}} + \Delta {\text{S/2}}.{3}0{\text{3R}}$$

5 $$\Delta {\text{G}} = \Delta {\text{H}} - {\text{T}}\Delta {\text{S}}$$

where R is the universal gas constant having a value of 8.314 J K^−1^ mol^−1^ and K_b_ is the binding constant. Slope and intercept obtained from the graph of log K_b_ versus 1/T (Fig. 8) represent the ΔH and ΔS. Change in enthalpy (ΔH) and change in entropy (ΔS) both defines the Gibbs free energy (ΔG) using the Eq. (5). The calculated thermodynamics parameters are summarised in Table 2.

**Table 2:** shows the calculated thermodynamics parameters of BSA with MiADMSA at different temperatures.

System	Temperature (K)	ΔH (kJ mol^−1^)	ΔS (J mol^−1^ K^−1^)	ΔG (kJ mol^−1^)
BSA-MiADMSA	298	4.50	0.019	− 1.162
	303			− 1.257
	308			− 1.352
	318			− 1.542

If the value of ΔH > 0, ΔS > 0 signifies the hydrophobic interactions, ΔH ˂ 0, ΔS ˂ 0 signifies the role of Van der Waals forces and hydrogen bond interactions, and if ΔH ˂ 0, ΔS > 0 signifies the electrostatic interactions^48^. From the calculated thermodynamic data negative ΔG indicates that our system follows a spontaneous binding process. The positive ΔH and ΔS indicate that hydrophobic forces play an important role in the binding of BSA with MiADMSA and the process of binding is endothermic. The outcomes of this study are directly correlated with synchronous fluorescence experiments that hydrophobic forces were developed during the interaction of BSA with MiADMSA.

### Circular dichroism spectroscopic analysis

**Figure 9:**
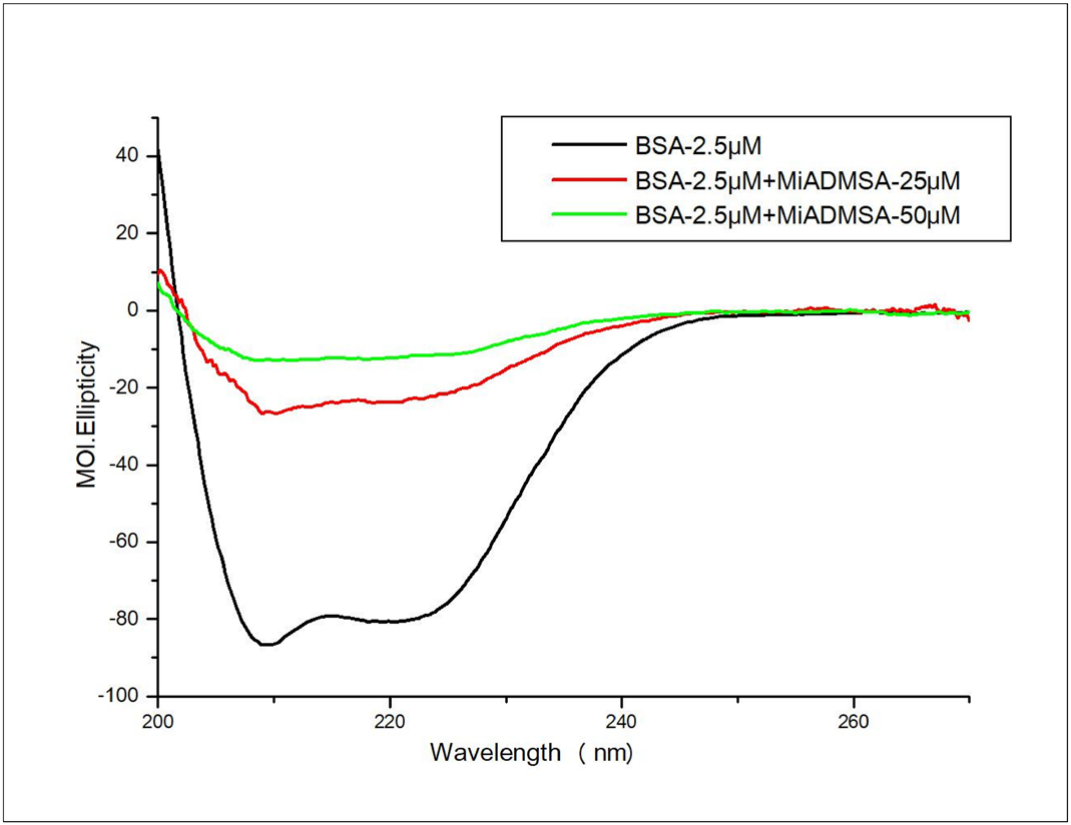
CD spectra of BSA alone (2.5 µM) and BSA with MiADMSA at 25 µM and 50 µM.

CD is widely used to elucidate the changes in the proteins secondary structure, and also be helpful in understanding the interaction of ligands or drugs with proteins^48^. The CD spectrum of BSA showed two negative peaks at 208 nm and 222 nm in the UV region which indicated n → π* electronic transition of the peptide bond, characteristic of the α-helix structure^22^. Due to this conformational change, the hydrophobic cavities get exposed and the microstructures around the aromatic amino acid residues were disturbed. The conformational changes of BSA upon successive addition of MiADMSA were evaluated using CD spectroscopy. Figure 9 depicted the CD spectra of BSA alone (2.5 µM) and in the presence of MiADMSA at 25 µM and 50 µM concentration. We found that adding 25 µM concentrations of MiADMSA in BSA causes significant changes in both the peaks. Further, the double concentration of MiADMSA (50 µM) led to more changes in the peaks. This indicates the binding of MiADMSA induced conformational change in the secondary structure of BSA in terms of α helix, sheet, and turn as shown in Table 3. The CD spectrum of BSA alone has shown helicity of 56.8% which was changed to 50.8% at 1:10 ratio of BSA:MiADMSA inferred that in presence of MiADMSA, β helical structure of the protein was disrupted. Further addition of 50 µM of MiADMSA in BSA causes significant changes in helical content and changed in sheet content also. This indicates the binding of MiADMSA induced conformational change in the secondary structure of BSA as shown in Table 3. Due to this conformational change, the hydrophobic cavities get exposed and the microstructures around the aromatic amino acid residues were disturbed. Thus, it could be attractive to conclude that MiADMSA caused a significant change in the secondary structure of BSA. This finding can be correlated with the fluorescence and UV-absorption studies in which we have observed a significant decrease in the fluorescence intensity of BSA which might be due to binding with MiADMSA.

**Table 3:** Alpha helical content and other parameters for secondary structure estimation of BSA and BSA with MiADMSA.

System	Helix (%)	Sheetx (%)	Turns (%)	Otherx (%)
BSA (2.5 µM)	56.8	0.0	0.0	0.0
BSA + MiADMSA (25 µM)	50.8	6.0	0.0	0.0
BSA + MiADMSA (50 µM)	45.6	11.2	0.0	0.0

### FT-IR spectral analysis

**Figure 10:**
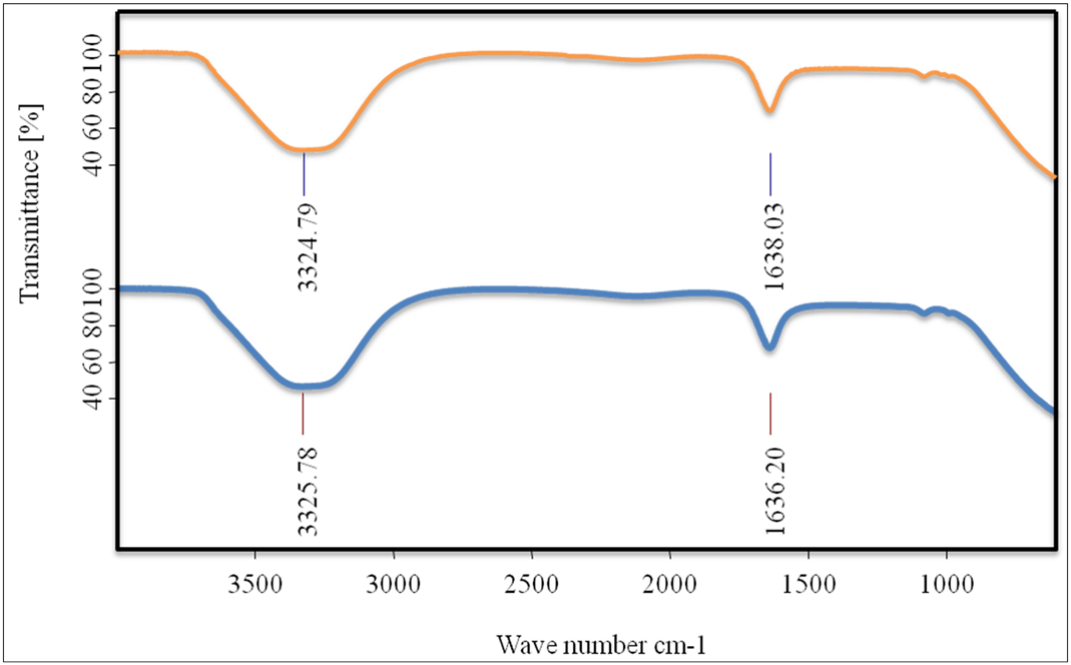
FT-IR spectra of free BSA (blue) and complex of BSA-MiADMSA (red).

FT-IR approach gives information about the conformational changes in the secondary structure of protein on interaction with drug^49^. BSA consists of various amino acids that are linked together via peptide bond to form a protein. If any ligand or drug bind to protein leads to alteration in the structure of the protein. All the IR spectra are recorded from 500 up to 4000 cm^−1^ wavenumber (Fig. 10)^23^. For the protein (BSA), in the case of amide-I, the vibrations due to N–H (bending) and C=O (stretching) are in the frequency range of 1500 to 1700 cm^−1^ and the vibration at a frequency near 3300 is due to N–H stretching. Moreover, in the case of amide-II, the vibration due to C–N stretching is medium in intensity. So, the amide-I peak provides more data about the alteration in the structure of the protein which is important in contrast to amide-II^17^. As shown in figure (Fig. 10) it clearly describes the importance of amide-I over the amide-II in which the two peaks at 1636 cm^−1^ and 3326 cm^−1^ in free BSA is slightly shifted towards 1638 cm^−1^ and 3325 cm^−1^ due to MiADMSA bind to BSA proposing that there is alteration in conformation of BSA due to interaction with MiADMSA.

### Molecular docking analysis

**Figure 11:**
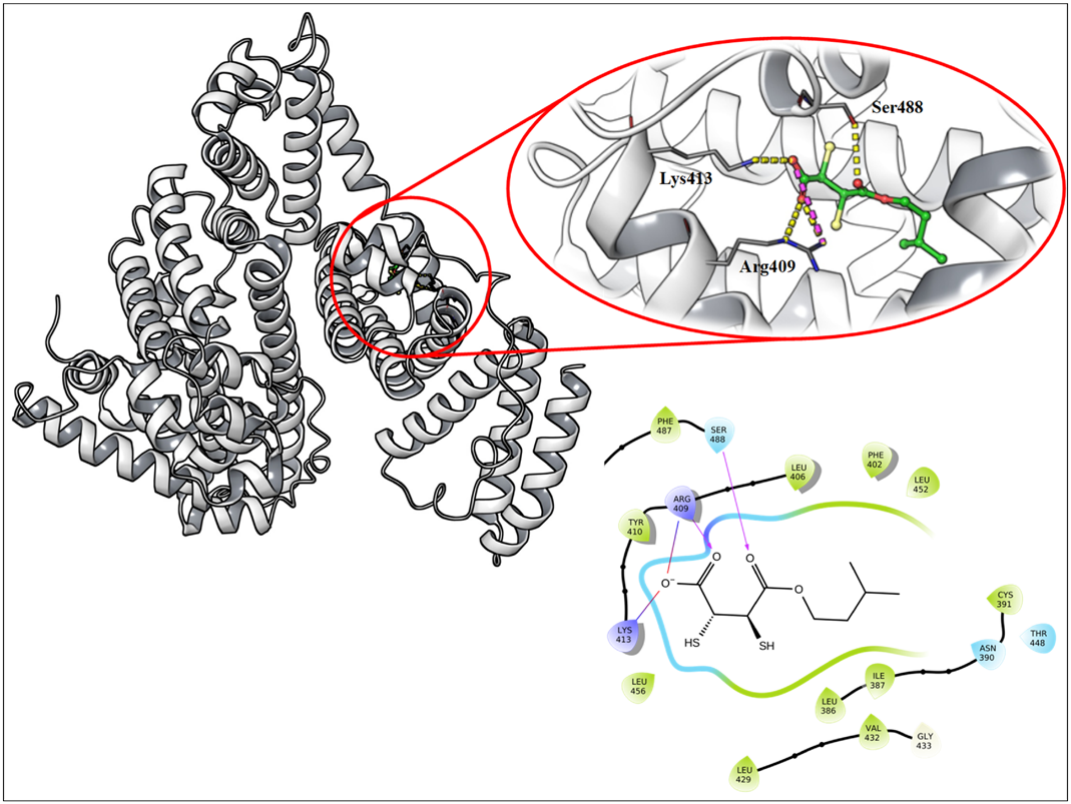
The docked pose of MiADMSA at ibuprofen binding site (SiteII_a_) of BSA (Desmond Academic license 2018-4, https://www.schrodinger.com/citations#Desmond).

Ibuprofen can bind at two different sites in BSA, docking was performed at both sites. However, we got good results only for one site and another site was not showing a better docking pose. However, we have performed simulation for both the docked poses to understand the binding modes of MiADMSA at two different sites. Docking scores for both the sites were given in the Table 4. Docking poses for MiADMSA at site II_a_ (Fig. 11) and II_b_ was provided in the supplementary file (S1). Docking result shows that negatively ionizable carboxylic group tends to form hydrogen bond as well as ionic interaction with Lys 413 as well as Arg 409. The hydrophobic tail of MiADMSA was placed nicely in the protein hydrophobic pocket (Fig. 11).

**Table 4:** Docking results of MiADMSA at two different binding sites.

S.no	Glide docking score (Kcal/mol)	Glide model score (Kcal/mol)	Interacting residues
1.*	− 6.42	− 66.77	Arg 409 and Ser 488, Lys 413, Leu 386, Ile 387, Leu 406 and Phe 408
2	− 3.80	− 30.23	Asn 44, Lys 20, Leu 24, Leu 138, Val 40

*Figure  11 docked pose.

Docking results show the binding pose of MiADMSA at site II_a_ of the ibuprofen binding site identified from ESA was showing better docking score than other sites. Hydrogen bond interaction observed between Arg 409 and Ser 488 was stable for 200 ns of MD simulation. Lys 413 was also involved in H-bond interaction but vanishes during the simulation. Other than H-bond interactions the hydrophobic tail of MiADMSA was well settled inside a hydrophobic pocket of protein (Leu 386, Ile 387, Leu 406, and Phe 408). The docked pose of MiADMSA-BSA at site II_b_ was shown in the supplementary file (S1 Fig. 2).

### Molecular dynamic analysis

**Figure 12:**
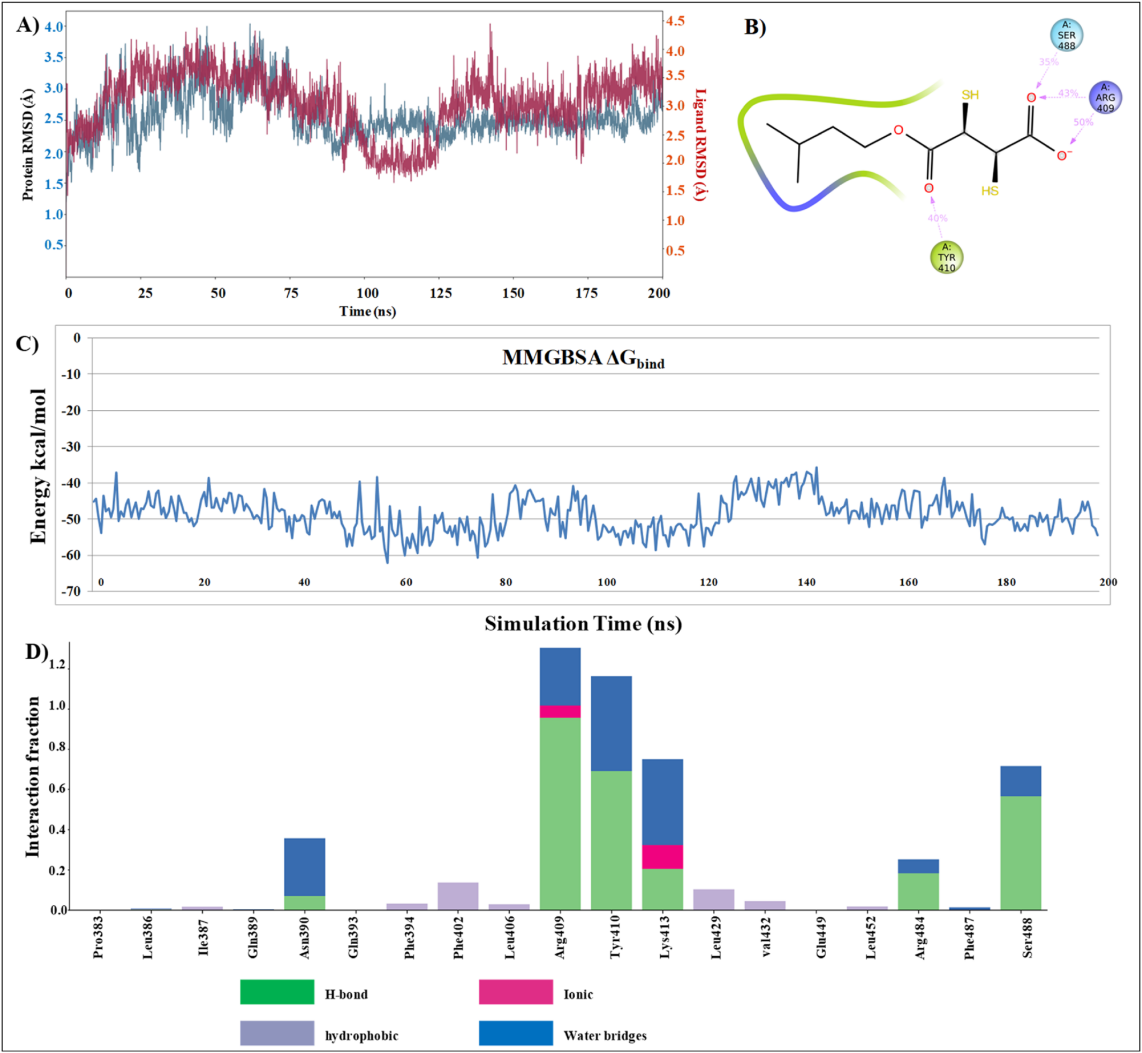
Molecular dynamics result for site II_a_ (**A**) RMSD of protein and ligand (**B**) BSA-MiADMSA interaction diagram during 200 ns of MD simulation (**C**) Binding free energy calculation on 200 ns of MD trajectory (**D**) Interacting residues with their interaction fraction throughout the simulation (Desmond Academic license 2018-4, https://www.schrodinger.com/citations#Desmond).

BSA-MiADMSA docked complex was simulated for 200 ns in Desmond 2018-4 to understand the protein–ligand stability. Post simulation analysis of trajectory revealed that the complex is stable with minimal deviation in RMSD and the ligand was able to make H-bond interaction with the protein (Fig. 12). RMSD of both protein–ligand complexes as well as a ligand is not deviating more than 3.2-Å. The hydrophobic tail of MiADMSA was well settled inside the hydrophobic region of BSA. Although we can observe that there is a decrease in the RMSD plot during 100 ns to 125 ns of MD simulation. However, the change was very minor and no major structural change was observed in the protein structure. We have also performed the MMGBSA binding free energy calculation in the complete MD trajectory and data was plotted with time on X-axis and ΔG_bind_ at Y-axis. Binding free energy calculation also provides support to our MD data and shows that there was no major fluctuation in protein–ligand conformation. With support to our experimental data, computational data provide the molecular details of MiADMSA binding with BSA at the ibuprofen binding site.

**Figure 13:**
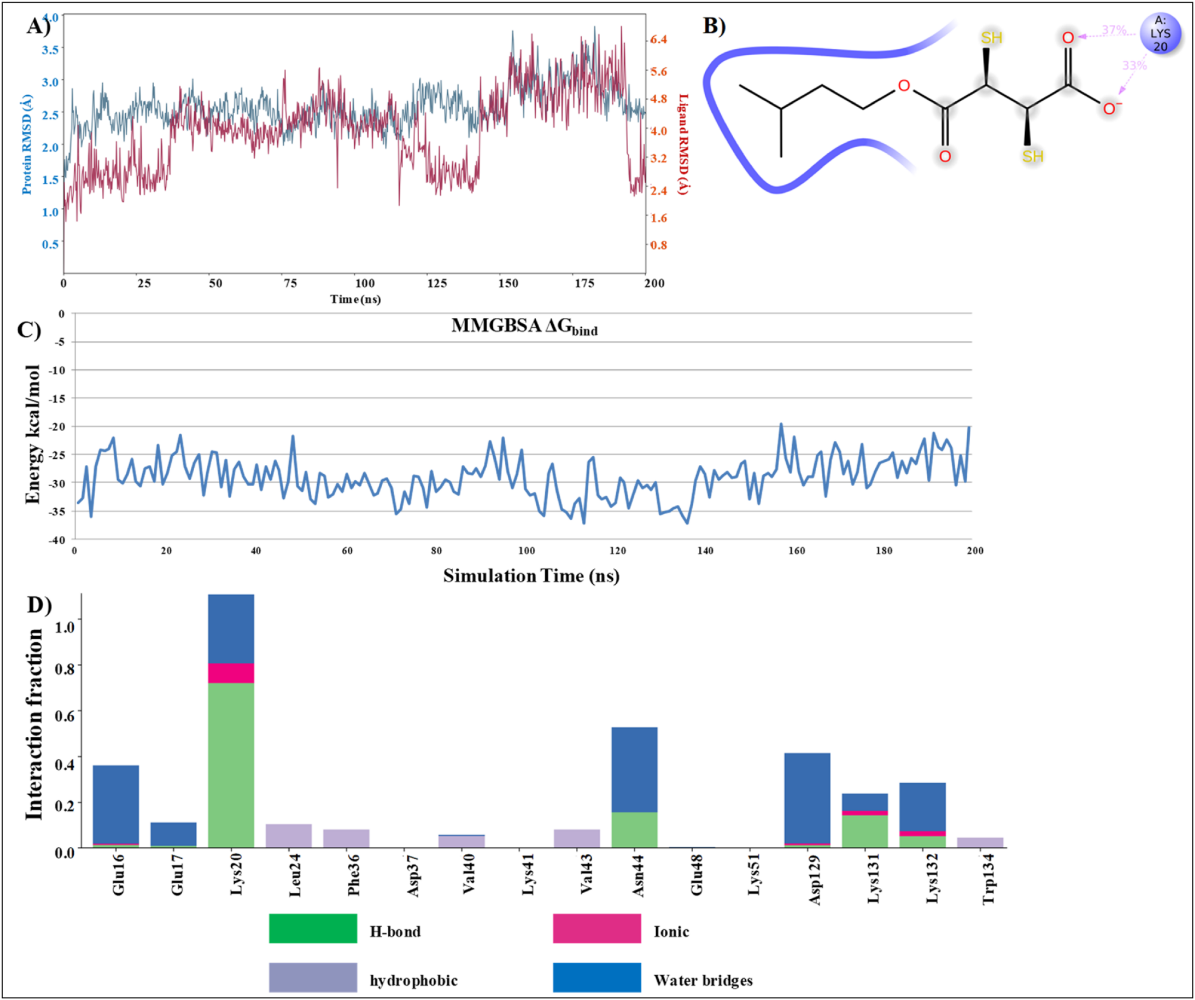
Molecular dynamics result for site II_b_ (**A**) RMSD of protein and ligand (**B**) BSA-MiADMSA interaction diagram during 200 ns of MD simulation (**C**) Binding free energy calculation on 200 ns of MD trajectory (**D**) Interacting residues with their interaction fraction throughout the simulation (Desmond Academic license 2018-4, https://www.schrodinger.com/citations#Desmond).

As compared to site II_a_ there is more fluctuation in protein RMSD in site II_b_ docked pose of MiADMSA. Binding free energy was also low in the case of site II_b_, possible reasons could be the hydrophobic interactions are much favored in site II_a_ as compared to site II_b_. The hydrophobic tail of MiADMSA was oriented towards a positively ionized pocket. In terms of hydrogen bonding, only Lys 20 was able to make a stable interaction with the ligand, and the rest of the interactions did not last for long, which could a possible reason for disturbance in protein RMSD (Fig. 13). A comparison of docked pose and last snapshot from MD was shown in the supplementary file (S1 Fig. 3).

## Conclusion

MiADMSA is a potential arsenic chelator which could be developed as an antidote for the treatment of  poisoning cases. In this study, we have used various spectrophotometric methods like Fluorescence spectroscopy, UV–Visible spectroscopy, FT-IR spectroscopy, and computational methods like molecular docking and molecular dynamics to analyze the binding interactions between the BSA and MiADMSA for understanding the pharmacokinetics and pharmacodynamics properties of the drug. Fluorescence emission analysis showed the fluorescence quenching of BSA upon binding with MiADMSA was dynamic in nature. The binding process is spontaneous and hydrophobic interactions are responsible for the complex formation. Moreover, site marker studies show the MiADMSA binds at site-II of BSA. CD spectroscopy, 3D fluorescence, FT-IR, and UV–Visible spectroscopy studies describe the conformational alterations in the secondary structure of BSA on binding with MiADMSA. Computational work was exclusively performed at the ibuprofen binding site and was further supported with 200 ns of MD simulation followed by binding free energy calculation. Docking studies at different binding sites in BSA also shed light that BSA tends to binds at the same binding site where ibuprofen binds. Although BSA possesses multiple binding sites and several crystal structures that provide evidence of binding of the same drug at different sites in BSA, an efficient method needs to be developed to provide a better understanding of drug binding with BSA. This work is useful in understanding the ligand–protein interactions and this study is also helpful in describing a lot of information about its storage, transportation, and metabolism of upcoming active molecules.

## Supplementary Information

Supplementary Information 1.
