# Supplementary material for: Interaction study of monoisoamyl dimercaptosuccinic acid with bovine serum albumin using biophysical and molecular docking approaches
Source: Sci Rep. 2021 Feb 18;11:4068. doi: 10.1038/s41598-021-83534-0 (PMC7892868; doi:10.1038/s41598-021-83534-0)
Supplement: Supplementary file 1 — Supplementary Information 1. [file 41598_2021_83534_MOESM1_ESM.docx]

**Supplementary File**

**Interaction Study of Monoisoamyl Dimercaptosuccinic Acid with Bovine Serum Albumin using Biophysical and Molecular Docking Approaches**

Ashima Thakur^1^, Jayant Patwa^2^, Suyash Pant^3^, Abha Sharma^1^*and SJS Flora^2^*

^1^Department of Medicinal Chemistry, National Institute of Pharmaceutical Education and Research, Raebareli, India

^2^Department of Pharmacology and Toxicology, National Institute of Pharmaceutical Education and Research, Raebareli, India

^3^Department of Pharmacoinformatics, National Institute of Pharmaceutical Education and Research, Kolkata, India

Correspondence:

Dr. Abha Sharma

Associate Professor

Department of Medicinal Chemistry

National Institute of Pharmaceutical Education and Research,

Bijnor-Sisendi Road, Sarojini Nagar, Near CRPF Base Camp,

Lucknow (U.P.) - 226002

E-mail: [abha.sharma@niperraebareli.edu.in](mailto:abha.sharma@niperraebareli.edu.in)

Dr. SwaranJeet Singh Flora

Director

National Institute of Pharmaceutical Education and Research,

Bijnor-Sisendi Road, Sarojini Nagar, Near CRPF Base Camp,

Lucknow (U.P.) - 226002

E-mail: [sjsflora@hotmail.com](mailto:sjsflora@hotmail.com)

**Fig. 1** the fluorescence quenching of BSA (5 µM) with increasing concentration of MiADMSA (0-128 µM) at different temperatures (303 K, 313 K, 318 K)

**Fig. 2** Docked pose of MiADMSA at site II_b_

**Fig. 3** **Comparison** of docked pose and last MD snapshot of MiADMSA-BSA **A)**For site II_a_ **B)** For site II_b_
